# The Impact of Pulmonary Rehabilitation in a Case of Acute Respiratory Distress Syndrome With Bronchopneumonia: A Case Report

**DOI:** 10.7759/cureus.32671

**Published:** 2022-12-18

**Authors:** Roshni R Nandanwar, Rajat Singh, Samruddhi M Karanjkar, Ritika S Bhagwani

**Affiliations:** 1 Physiotherapy, Ravi Nair Physiotherapy College, Datta Meghe Institute of Medical Sciences, Sawangi, Wardha, IND

**Keywords:** pulmonary rehabilitation, physical therapy, physiotherapy, pneumonia, ards

## Abstract

Inhaled bacteria and viruses could cause pneumonia (from the Greek word pneuma, which means "breath"), which is an infection and inflammation of the bronchioles and alveoli in the lower respiratory tract that can be fatal. The condition's typical symptoms include excruciating chest pain and a persistent cough that produces thick mucus. Of patients in emergency medicine units around the world, 10% have acute respiratory distress syndrome (ARDS). A 35-year-old male patient stated having a fever for 12 days, a cough with expectoration for two days, and trouble breathing at rest when he arrived at the medical emergency unit. Following the examination, blood investigation, urine examination, and X-ray were done suggestive of bronchopneumonia and acute respiratory distress syndrome. The patient was assessed using a range of outcome measures on the assessment day, and the same variables were again assessed on the discharge and follow-up days. These outcome measures showed significant reduction in the severity of the cough and dyspnea. Also, the patient had markedly improved cough intensity, dyspnea (Modified Medical Research Council {MMRC}, grade 2), lung capacity, weakness, and quality of life (QoL) because of our well-organized pulmonary rehabilitation. It is safe to assume that a thorough strategy like ours will lead to an improvement in the patient's respiratory health.

## Introduction

Acute respiratory distress syndrome (ARDS) accounts for 10% of all patients in critical care units around the globe [1]. Acute respiratory distress syndrome may be brought on by infectious conditions such as pneumonia with or without sepsis or by a number of pathogens, including pneumococci, influenza viruses, coronaviruses, and malaria [2]. Moreover, ARDS is linked to a number of noninfectious conditions, including pancreatitis, aspiration of gastric contents, near drowning, traumatic chest contusion, multiple traumas, inhalation burns, and multiple blood transfusions [3]. Poor oxygenation, respiratory infiltrates, and an early onset are hallmarks of the possibly deadly illness. The illness is linked to major alveolar destruction and capillary endothelial dysfunction on a microscopic level [2]. ARDS is marked by bilateral alveolar infiltrates, severe developing hypoxia, and no signs of cardiogenic pulmonary congestion. It occurs within seven days after the initial episode. Berlin's criteria (2012) define ARDS on the basis of partial pressure of oxygen (PaO_2_)/fraction of inspired oxygen (FiO_2_) ratio into mild, moderate, and severe categories. They advocate the use of continuous negative airway pressure (CNAP) or positive end-expiratory pressure (PEEP) that exceeds 10 mm Hg.

Lung vascular damage is the most essential and crucial element causing ARDS. There is significant evidence that, even in the normal range of lung vascular pressure, an increase in lung vascular permeability mainly impacts lung microcirculation and contributes to the development of protein-rich pulmonary deem [4]. There are several routes that might harm the lung endothelium, but neutrophil-dependent lung damage could be the most extensively researched one. Neutrophils are the evaluative terminal mechanism of lung damage across several experimental scenarios, including acid-induced lung injury and transfusion-associated lung injury [2-5,6]. Although the idea of neutrophil-dependent lung problems is significant, it must also be understood in the context of neutrophils' crucial contribution to host defense, notably against bacterial infection. As a result, whereas neutrophil depletion can partially or completely prevent lung damage in animal models, it clearly compromises innate immunity [7]. Any physical therapy program in a critical area should generally apply cutting-edge, inexpensive treatment modalities to reduce a patient's reliance on a ventilator, improve residual function, minimize the need for repeated hospitalizations, and improve the patient's quality of life (QoL) [8]. Patients with severe acute respiratory distress syndrome who require mechanical ventilation are more prone to experience muscle weakness and exercise intolerance; as a result, early rehabilitation therapy that starts during the acute illness stage is essential for improving their physical performance. Early physical treatment can act as a rehabilitation bridge for acute respiratory distress syndrome patients.

## Case presentation

A 35-year-old male patient presented with complaints of a fever for 12 days, a cough with expectoration for two days, and difficulty in breathing at rest, as well as while doing activities (Modified Medical Research Council {MMRC}, grade 4), when he arrived at the medical emergency unit. Following the examination, blood investigations and urine examination findings were normal, and an X-ray was done that suggested bronchopneumonia and acute respiratory distress syndrome. He has been instructed to seek further treatment at physiotherapy and advised admission to the medical ICU. The patient was conscious and alert with a heart rate of 90 beats per minute, a respiration rate of 24, a blood pressure of 120/70 mm Hg, a weight of 54 kg, and an oxygen saturation of 94% on room air. Observation findings revealed normal chest shape but limited chest movement on both sides and increased usage of accessory muscles, which increased the strain of breathing. On palpation, diminished chest expansion on both sides was seen. Air entry was reduced in both the lower zones during auscultation. No other previous illness or surgical history was found. The patient was on medication such as Augmentin 625 mg twice per day (BID), tablet pantoprazole 40 mg, Montair-LC Tablet, and nebulization with Budecort along with Duolin.

Investigatory findings

**Figure 1:**
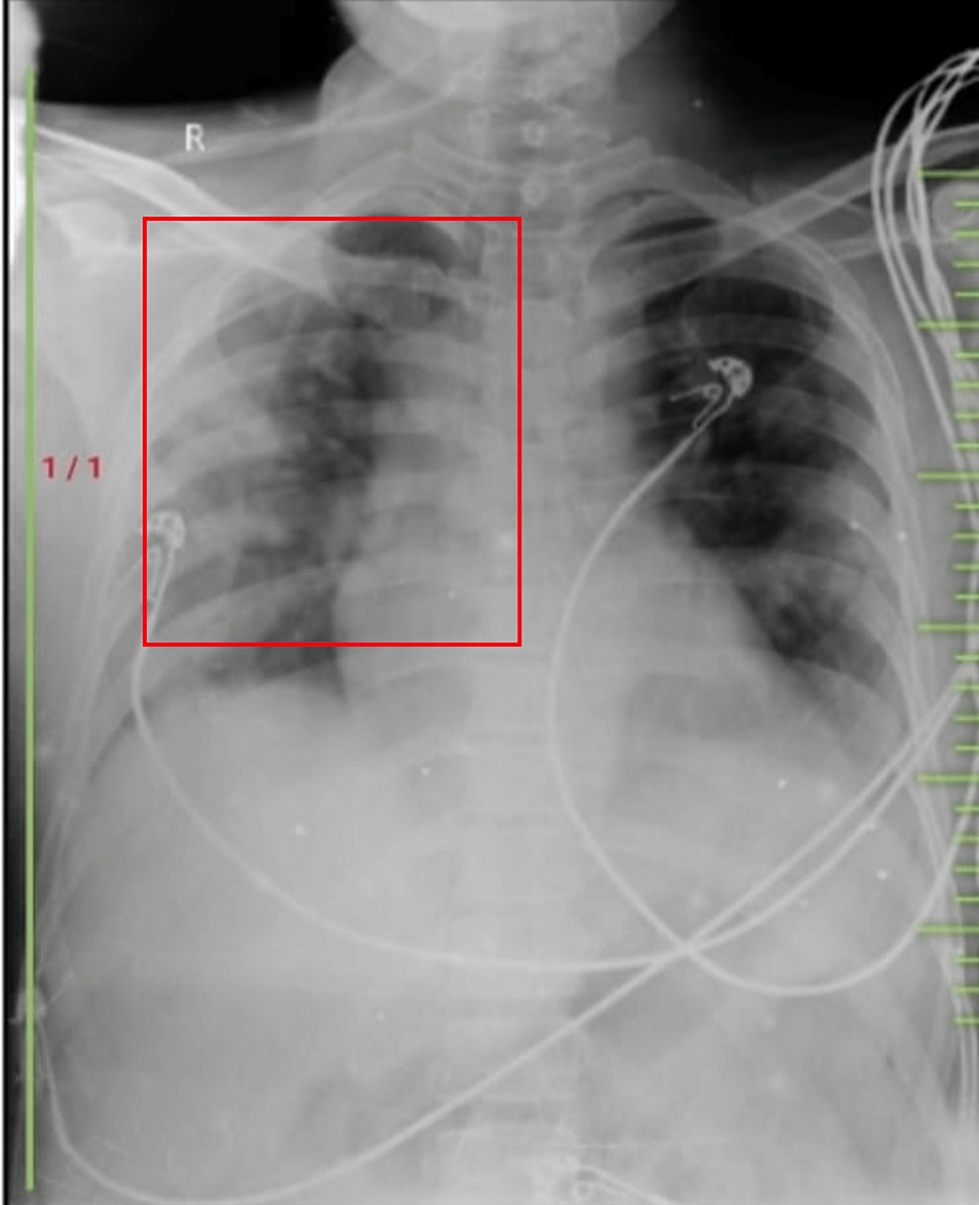
Hyper-translucency of the lung field with bronchovesicular markings present and hilar marking present (right side)

Chest X-rays were carried out in posteroanterior view (Figure 1), which shows hyper-translucency of the lung field with bronchovesicular markings present and hilar marking present (right side).

Therapeutic Interventions

**Figure 2:**
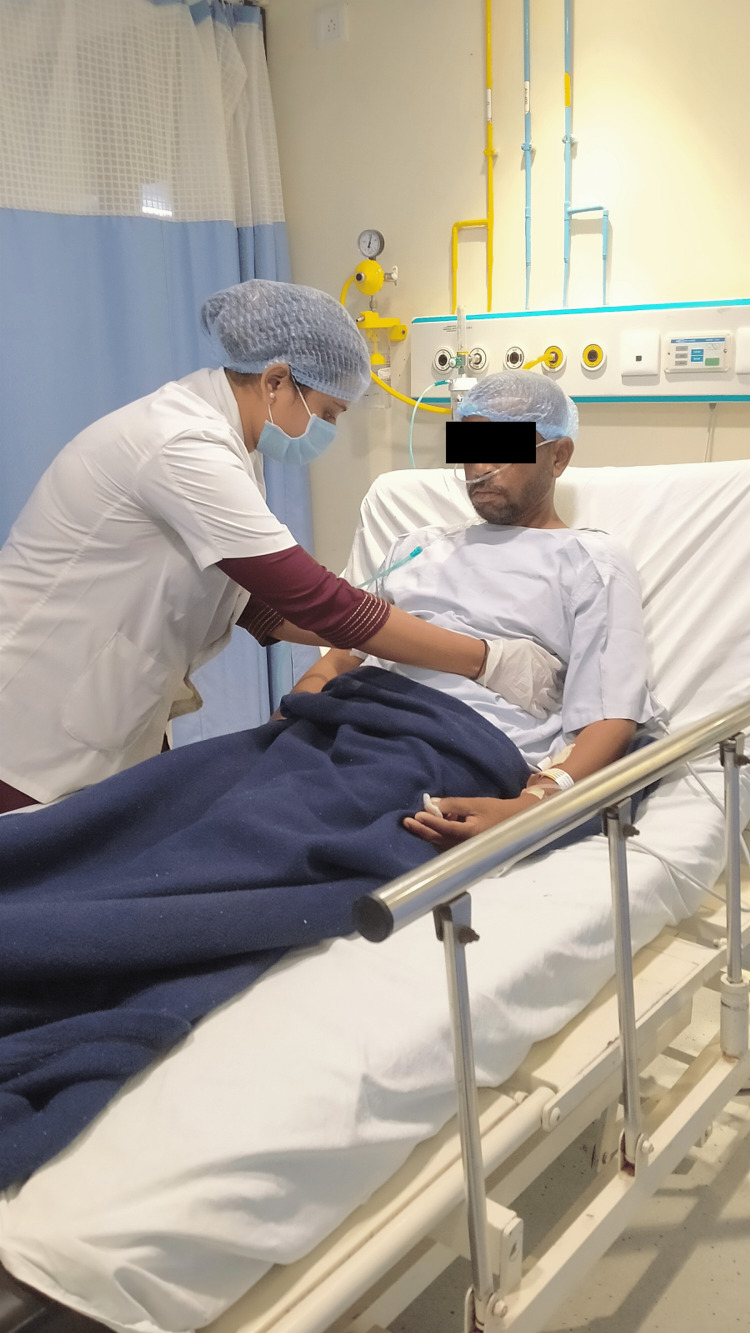
Segmental expansion in lower zones in both side

**Figure 3:**
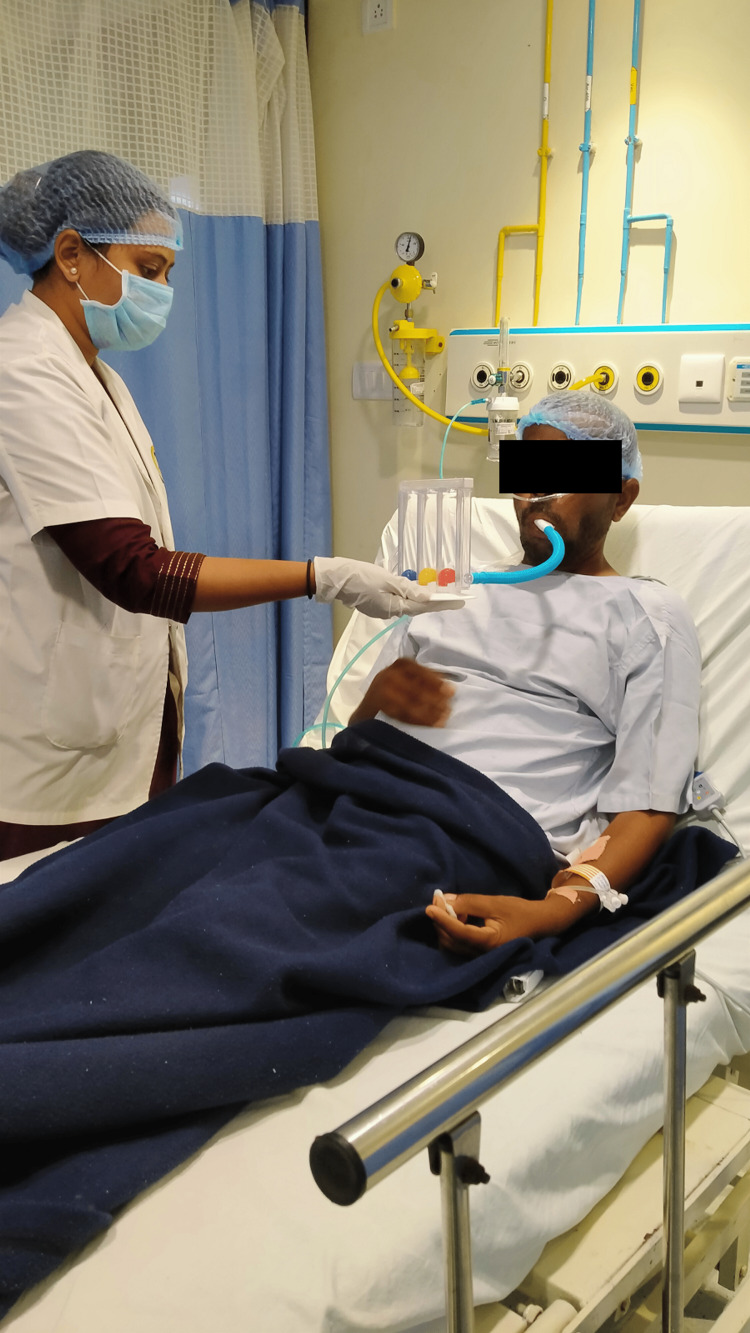
Incentive spirometer

The patient was managed using an interdisciplinary approach, which helped him recover quickly. By enhancing gaseous exchange and lung air entry, our main goal was to improve hypoxemia and relieve dyspnea. The following intervention is shown in Figure 2, i.e., segmental expansion for lower zones, and Figure 3 shows incentive spirometer.

Physiotherapy treatment goals and management

The physiotherapy treatment was split. First, the patient was informed of his condition, and informed consent for treatment procedure was obtained in order to instill the value of adhering to the prescribed course of action. We then focused on removing obstructive tracheobronchial secretions in order to reduce airway resistance and improve work of breathing. Aerosol therapy and nebulization with Budecort along with Duolin were given three times a day for a week to loosen the mucus and improve the air passage into the lungs. Outcome measures were taken during assessment and on the last day of treatment (after three weeks) as shown in Table 1. Complete intervention is given as shown in Table 2.

**Table 1:** Outcome measures (pretreatment)

Scales	Scores
Numerical Pain Rating Scale score (on walking)	8/10
Modified Medical Research Council dyspnea scale	Grade 4
WHO Quality of Life-Physical Performance	52/100
Emotional intelligence	71/100
Social interaction	85/100
Environmental performance	68/100

**Table 2:** Intervention reps: repetitions

Serial number	Intervention objectives	Clinical intervention	Treatment protocol
1	Patient education	Explain the treatment protocol	Explain the treatment protocol at the beginning of treatment
2	Improving bed mobility and avoiding extended immobility and prone positioning	Transitional training in bed under observation to prevent bed sores and keeping the patient in prone position to improve oxygenation	Positioning of the patient in prone should be done for a minimum of six hours daily.
3	To restore standard activities of daily living	Walking at your own pace down a 30 m corridor	Ambulation under the supervision of a therapist two times a day
4	To improve respiratory rate and breathing pattern	Deep breathing exercise, segmental expansion, and diaphragmatic breathing exercise	Segmental breathing for lower zones and diaphragmatic breathing 10 reps in one set three times a day
5	To enhance lung capacities and volumes (functional residual capacity) by evaluating through a spirometer	Utilizing a flow-oriented incentive spirometer	A three-second hold on 600 cc with 10 reps
6	To keep joints mobile and healthy	Bilateral active range of motion workouts for the upper extremities and lower extremities	At first, perform 10 reps in one set twice day. Afterward, perform 10 reps in two sets three to four times per day for three weeks
7	To improve posture	Posture adjustment	He proactively corrected his posture by not slouching as a result of passive input from family members and self-feedback

Follow-up

After one week of pulmonary rehabilitation, he was able to perform activities of daily living with only minimal assistance, and symptoms such as pain and shortness of breath were reduced. The patient developed self-assurance and a positive outlook in life. Two sessions each day of the exercise program were conducted in the hospital for four weeks. After four weeks, the patient's lung exercise tolerance capacity showed just a slight improvement. The patient was able to walk easily as the patient's dyspnea grade changed from 4 to 2. The patient maintained the oxygen saturation of 96% on room air and was able to perform incentive spirometer with 900 cc with 10-second hold. Outcome measures posttreatment are mentioned in Table 3.

**Table 3:** Outcome measure (posttreatment)

Scales	Scores
Numerical Pain Rating Scale score (on activity)	5/10
Modified Medical Research Council dyspnea scale	Grade 2
WHO Quality of Life-Physical Performance	89/100
Emotional intelligence	88/100
Social interaction	85/100
Environmental performance	85/100

## Discussion

Because of widespread alveolar collapse, which causes hypoxia and a ventilation-perfusion mismatch, the fundamental problem with acute respiratory distress syndrome is oxygenation rather than ventilation [9]. Significantly less of the normal lung's capacity is present, and alveolar compliance and time constants vary substantially [10]. The goal of our pulmonary rehabilitation was to focus on and treat all of the patient's recent problems, as well as any future problems such as lung collapse and fibrosis. The physiotherapy management consisted of pulmonary rehabilitation and psychological counseling. The primary goal of the pulmonary rehabilitation interventions was to improve the quality of life (QoL) by lowering cough frequency, enhancing ventilation, and decreasing sleep disturbance. Incentive spirometer, thoracic expansion exercises, segmental breathing, and diaphragmatic exercises were all utilized to improve lung capacity. The following outcome measures were used to assess the effect of the patient's treatment: Modified Medical Research Council (MMRC) dyspnea scale, Numerical Pain Rating Scale (NPRS), and the WHO Quality of Life (WHOQOL) questionnaire.

On the first day of the physiotherapy examination, the day of discharge, and the day of follow-up, all the scales and questionnaires were examined. The variation in scores during these three days indicated substantial progress on each measure. The only obstacles faced in managing this patient were the patient's illiteracy and the family's lack of participation. In a quasi-experimental design utilizing an ICU mobility team, some studies established the safety and viability of early activity in the ICU, starting at the admission of medical ICU patients on mechanical ventilation [11]. This early activity strategy was substantially related to shorter stays in the ICU and hospitals but was not associated with an increase in future related problems [12]. In our study, we have even focused on future complications such as pulmonary function decline, respiratory muscle weakness, and depression and managed the patient by improving his overall health status such as improving exercise tolerance, maintaining the strength of respiratory muscles, and improving lung capacity to prevent further complications. In a study with pulmonary rehabilitation that includes breathing exercises, aerobic training, and simplified strength training, prior research showed that individuals with ARDS experience both temporary and long-term detrimental limits in physical function [13]. According to several studies, pulmonary rehabilitation can aid in enhancing exercise capacity, sleep quality, depression, and quality of life in terms of health [14-16]. For the treatment of severe ARDS with persistent hypoxemia, a specific standard of care is essential [17]. Our study will provide the way for pulmonary rehabilitation programs to be introduced in the majority of hospitals and community centers, and patient awareness of their advantages will lead to increased involvement and improvements in functional characteristics and quality of life [18]. Few articles showed complete functional recovery of acute respiratory distress syndrome patient with pulmonary rehabilitation [19,20].

## Conclusions

Respiratory muscle weakness and exercise intolerance are more likely to occur in patients with severe acute respiratory distress syndrome who need mechanical breathing, Therefore, early rehabilitation therapy that begins during the acute sickness stage is essential for enhancing their physical function. Early physiotherapy for acute respiratory distress syndrome acts as an important adjunct to recovery. However, the patient had markedly improved cough intensity, dyspnea, pulmonary capacity, weakness, and general quality of life because of our well-organized pulmonary rehabilitation. It is safe to assume that a thorough strategy like ours will lead to an improvement in the patient's respiratory health.
